# Circular RNA PIP5K1A act as microRNA-552-3p sponge to regulates inflammation, oxidative damage in glucolipotoxicity-induced pancreatic INS-1 β-cells via Janus kinase 1

**DOI:** 10.1080/21655979.2021.2022076

**Published:** 2022-02-19

**Authors:** Lei Ren

**Affiliations:** Department of Endocrinology, First Affiliated Hospital of Zhengzhou University, Zhengzhou, Henan, China

**Keywords:** Type 2 diabetes, INS-1Eβ-cell, circular RNA PIP5K1A, microRNA-552-3p, Janus kinase 1-STAT3 pathway, inflammation, oxidative damage, apoptosis

## Abstract

Elevated level of glucolipotoxicity induces the loss of pancreatic β-cells functions and plays an important role in the development of type 2 diabetes (T2DM). Previous studies have indicated the importance of developing therapies against T2DM, while circular RNA (circRNA) has gained attraction as a modulator of pancreatic β-cell function. In the present study role of circPIP5K1A in dysfunctional β cells and mouse pancreas was comprehensively analyzed. INS-1E, as it has close similarity with naïve pancreatic β-cells, and clinical samples of T2DM patients were used to investigate the effect of circPIP5K1A, miR-552-3p, and Janus kinase 1 (JAK1). While, INS-1E cells were exposed to PAHG conditions (0.5 mM palmitic acid and 28 mM glucose) as studies have suggested that increased level of fatty acid and glucose resulted in autophagy activation of pancreatic β-cells that leads to T2DM. Key player of JAK1-STAT3 pathway and the level of Reactive Oxygen Species, inflammatory factors, and insulin secretion was detected to analyze the of the active association of circPIP5K1A, miR-552-3p with JAK1pathway. Our study has revealed the elevated level ofcircPIP5K1A and JAK1, but reduced level of miR-552-3pin the serum of T2DM patients. Furthermore, we also found that reduced expression ofcircPIP5K1A leads to decreased rate of inflammation, oxidative damage and apoptosisinINS-1E cells induced by glucolipotoxicity. CircPIP5K1A was available to competitively combine with miR-552-3p, while whose direct target was JAK1. In conclusion, our study suggested a novel involvement of circPIP5K1A in a cross talk between miR5523p/JAK1/STAT3 pathways in β-cells as a new therapeutic target for T2DM.

## 1 Introduction

Type 2 diabetes mellitus (T2DM) is a long-term illness featured via insulin resistance and pancreatic β-cell dysfunction [1,2]. Glucose and fatty acid provide energy for proper functioning of the cells but their elevated level resulted in β-cell dysfunction which may leads to increase rate of β-cell apoptosis, inhibition of glucose-stimulated insulin secretion, which ultimately reduced the β-cell mass and induce T2DM [2,3]. Additionally, the fusion of glucolipotoxicity enhanced the damaging effects of chronic elevations of glucose and fatty acids on pancreatic β cells [4,5]. Therefore, it is essential to investigate the molecular mechanisms of β-cell adaptation for designing the new therapies for T2DM.

Circular RNAs (circRNAs) are newly discovered endogenous non-coding RNA [6]. Meanwhile, circRNA forms a covalently closed loop with no free ends 3ʹ and 5ʹ [7,8]. Until now, circRNAs are famous for being extensively manifested in many tissues and eukaryotes and function in various biological and pathological processes [9]. For instance, circRNAs are supposed to be involved in β-cell dysfunction mediated by pro-inflammatory cytokines, thereby advancing type 1 diabetes Mellitus (T1DM) [10]. Circ-Tulp4 accelerates β-cell orientation to glucolipotoxicity via modulating soat1 [2]. CircPIP5K1A is a newly discovered circRNAs linked with cancer [8]. Li [11]*et al*. reported that circPIP5K1A is upregulated in T1DM patients. Nevertheless, the latent regulatory mechanism of circPIP5K1A in β cells of T2DM requires further elucidation.

MicroRNA (miRNA) is a subgroup of non-coding RNA (ncRNA). MiRNA particularly combines with the 3ʹ-untranslated region (UTR) of messenger RNA (mRNA), control gene at the post-transcriptional level, and takes part in development of all kinds of diseases [11–13]. Cell cycle is an important event in the process of cell proliferation while any abnormality in this process such as uncontrollable division leads to serious health conditions for instance in cancer [11]. Till the date number of miRNAs have been claimed to be involved in the development and progression of pancreatic cancer [14]. Furthermore, some studies have suggested that increased expression of miR-552-3p results in development and reoccurrence of ovarian cancer [15].

Other than cancer metabolism related diseases, such as glycolipid metabolism is one of the main concern among health-related problems around the world as lipid and glucose metabolism irregularities resulted in glycolipid metabolic disorders such as diabetes. Fan et al. [16] have suggested that miR-552-3p is the novel modulator of ameliorate hepatic glycolipid metabolic diseases by regulating LXRα- and FXR-related transcriptional activities in the nucleus. Ma *et al*. [17] also have suggested that miR-552-3p is a novel inhibitor with a dual repressive mechanism, which makes miRNA-552-3P a potential therapy against hyperlipidemia. Lei *et al*. [18] reported that miR-552-3p controls FXR’s and LXR’s transcriptional activity to mitigate liver glucose and lipid metabolism disorders. Janus kinase 1 (JAK1)-STAT3 signal pathway is a cytokine-induced signal transduction pathway found recently. JAK/STAT3 pathway consisted of tyrosine kinase-linked receptors, Janus kinases, and signal transducers and transcriptional activators [19]. This pathway participates in numerous momentous biological processes like immune modulation, differentiation, and apoptosis [20]. The studies of Francesca M Couto *et al*. [16] emphasized that Exenatide (Ex-4) protects β cells from cytokine-induced cell death via repressing JAK1-STAT1. Arianna Mazzoli*et al*. [21] announced that JNK1 ablation perfects the glucose tolerance of db/db mice via declining the loss of functional β cells in diabetes. However, whether miR-552-3p controls the β cells of T2DM through the JAK1-STAT3 pathway is still uncertain. These findings suggested that miR-552-3p may function as a possible target for metabolism-related diseases.

Objective of this study was to investigate the role and molecular mechanism of circPIP5K1A in β cells of T2DM and to investigate the link among circPIP5K1A, miR-552-3p with JAK1-STAT3 pathway. We investigated the correlation between circPIP5K1A and miR-552-3p, and circPIP5K1A and JAK1. Meanwhile, we also investigated the binding ability of circPIP5K1A and miR-552-3p, and miR-552-3p and JAK1 3ʹUTR. Finally, we illustrated the role of circPIP5K1A in regulating miR-552-3p and indirectly affecting the JAK1 expression.

## 2 Materials and methods

### 2.1 Clinical sample

Human blood serum samples of healthy and T2DM patients were collected from Harvest of First Affiliated Hospital of Zhengzhou University. According to the diagnostic criteria of WHO (1999), oral glucose tolerance test (OGTT) has been conducted, with fasting blood glucose (FPG) ≥7.0 mmol/L, and postprandial 2 h blood glucose (2 h PG) ≥11.1 mmol/L was conducted. Exclusion standards were patients bearing hypertension, malignant tumors, polycystic ovary syndrome, lung diseases, and autoimmune diseases; patients carrying other diseases impacting glucose metabolism were excluded, like hyperthyroidism, acute metabolic syndrome, acromegaly, and Cushing’s syndrome or other stress conditions and smokers. Inclusion of subjects with FPG < 6.1 mmol/L and 2 h PG < 7.8 mmol/L was considered as healthy controls. Quick freezing of the sample was done in liquid nitrogen and then the sample was stored at −80°C for later use. The project was approved via the Ethics Committee of First Affiliated Hospital of Zhengzhou University. Signed consent from all participants has been obtained before sampling.

### 2.2 Cell culture

The rat β cell line INS-1E was purchased from the National Cell Resource Center (Beijing, China). INS-1E cells was cultured and maintained in Roswell Park Memorial Institute (RPMI) 1640 medium consisting of glucose (11.1 mM), supplemented with 10% (v/v) fetal bovine serum (FBS), 100 U/mL penicillin, 100 µg/mL streptomycin, 1 mM sodium pyruvate, 10 mM 4-(2-hydroxyethyl)-1-piperazineethanesulfonic acid, and 50 μM 2-mercaptoethanol (all from Life Technologies, CA, USA). To induce glucolipotoxicity, INS-1E cells were exposed to palmitic acid (PA) and high glucose (PAHG) conditions for 24 h, which consisted of 0.5 mM PA and 28 mM glucose (both from Sigma-Aldrich, MO, USA) [22].

### 2.3 Cell transfection

CircPIP5K1A (si-circPIP5K1A#1, si-circPIP5K1A#2, and si-circPIP5K1A#3), miR-552-3p mimic/inhibitor, JAK1 elevation plasmid, and the corresponding negative control (NC) were obtained from RiboBio (Guangzhou, China). According to the manufacturer’s protocol, INS-1E cells have been transfected with these oligonucleotides for 48 hours by using Lipofectamine 2000 (Invitrogen, CA, USA) [14].

### 2.4 Cell counting kit (CCK-8) assay

Glucolipotoxicity of PAHG on INS-1E cells has been determined by CCK-8 (Beyotime Biotechnology, Shanghai, China) by following the manufacturer’s protocol. INS-1E cell has been cultured in 96-well plate with the cell density of 4000 cells/well and allowed to grow for next 4 days. Absorbance was read at 450 nm wavelength in a plate reader (DU650, Beckman Coulter, CA, USA). However, the cell viability graph has been plotted by using mean values [23].

### 2.5 Flow cytometry

Apoptosis rate of INS-1E cells was examined by flow cytometry by using annexin V-fluorescein isothiocyanate (FITC)/propidium iodide (PI) according to the (BD Biosciences, CA, USA) protocol. In short, INS-1E cells were cultured in 6-well plate for 4 days. Then the cells were harvested, rinsed off, and resuspended in PBS (phosphate buffer saline). Rate of apoptotic cell death was measured by usingAnnexin V-FITC and PI double staining. Reactive oxygen species (ROS) production was also examined via fluorescence-activated cell sorting (FACS) analysis. For the detection of ROS, the cells were harvested, resuspended in PBS and incubated with 5 μM DCFDA, and analyzed immediately by flow cytometry, as described previously [3].

### 2.6 Enzyme-linked immunosorbent assay (ELISA)

Total inflammatory cytokines tumor necrosis factor (TNF)-α and interleukin (IL)-6 production in the serum was detected by using ELISA kit according to the manufacturer’s protocol (Sigma-Aldrich) [24].

### 2.7 Glucose-stimulated insulin secretion determination

To determine the glucose-stimulated insulin secretion, INS-1E cells were cultured in RPMI-1640 (containing 0.5 mM PA) supplemented with KRB buffer and 28 mM glucose for 4 d. The insulin secretion was determined by strictly following the instructions of ELISA kit (Sigma, MO, USA) [24].

### 2.8 Reverse transcription quantitative polymerase chain reaction (RT-qPCR)

Total RNA was extracted from the INS-1E cells by using TRIzol reagent (Invitrogen)by using manufacturer’s protocol and quantified by spectrophotometry followed by reverse transcription of RNA into cDNA. RT-qPCR was performed via miScript SYBR-Green PCR Kit (Qiagen, Dusseldorf, Germany). The primer sequences of the target genes are listed in Table 1. U6 or glyceraldehyde-3-phosphate dehydrogenase (GAPDH) was used as a control. Determination of the result was via the 2^−ΔΔCT^ method [14].

**Table 1.** Primer sequence

Genes	Primer sequence (5ʹ – 3ʹ)
MiR-552-3p	Forward: 5ʹ-CCGCACAGGTGACTGGTTAGA-3ʹ
	Reverse: 5ʹ-GTGCAGGGTCCGAGGT-3ʹ
U6	Forward: 5ʹ-CTCGCTTCGGCAGCACA-3ʹ
	Reverse: 5ʹ-AACGCTTCACGAATTTGCGT-3’
CircPIP5K1A	Forward: 5ʹ-AGATTCCCTAACCTCAACCAGA-3ʹ
	Reverse: 5ʹ-CGAATGTTCTTGCCACCTGC-3ʹ
JAK1	Forward: 5ʹ-CATGGTGGAAGAGTTTGTGGAA-3ʹ
	Reverse: 5ʹ-CAGCTGTTTGGCAACTTTGAATT-3ʹ
GAPDH	Forward: 5ʹ-AAGGTCGGAGTCAACGGATTT −3ʹ
	Reverse: 5ʹ- CCATGGGTGGAATCATATTGG-3ʹ

### 2.9 Western blot

To elucidate the effect of PAHG on the INS-1E cells, the Western blot analysis has been performed. The INS-1E cells has been cultured and maintained in PRMI-1640 and exposed to PAHG conditions for the determined time period. After that, the cells were washed with PBS and trypsinized and incubated with protein lysis buffer for 30 minutes on ice. The samples were then centrifuged at 4°C for 10 min at 12000 rpm. Supernatant has been transferred to the new Eppendorf, and protein concentration was measured. Protein lysates were separated by using SDS-PAGE and PVDF technique of electrophoresis. PVDF membrane was blocked in 5% of BSA followed by incubation with relevant antibodies including primary anti-JAK1 (1: 1000), anti-p-STAT3 (1: 1000), anti-STAT3 (1: 1000), anti-GAPDH (1: 1000)), and then treatment with horseradish peroxidase-conjugated secondary antibody was visualization. However, all antibodies were obtained from Abcam (MA, USA) [11].

### 2.10 Subcellular separation

Subcellular separation has been performed by using PARIS kit (Life Technologies). In line with the manufacturer’s protocol, the cells were assigned to nuclear and cytoplasmic parts prior to proceeding with the RNA isolation procedure. In the meantime, GAPDH or U6 was considered cytoplasmic and nuclear controls [14].

### 2.11 The luciferase activity assay

To investigate the effect of PHAG condition on circPIP5K1A, miR-552-3p, and JAK1in INS-1E cells, luciferase assay has been performed. This pathway participates in numerous momentous biological processes like immune modulation, differentiation, and apoptosis. Prediction of the latent binding link among circPIP5K1A, miR-552-3p, and JAK1 was through the website of starbase (http://starbase.sysu.edu.cn). The 3ʹUTR sequence of circPIP5K1A or JAK1 consisting of miR-552-3p wild-type (WT) or mutant (MUT) binding sites was integrated into the pmirGLO vector (Promega, WI, USA) to form WT-circPIP5K1A or MUT-circPIP5K1A and WT-JAK1 or MUT-JAK1, separately. Briefly the transfection of miR-552-3p or mimic-NC has been done by using Lipofectamine 2000 (Invitrogen) by following the manufacturer’s protocol. After 48 h of transfection, luciferase activity was measured via dual-luciferase reporting kit (Promega) [19].

### 2.12 RNA-pull down assay

RNA-pull down assay has been executed by using pierce magnetic RNA-protein pull down kit (Sigma Aldrich). Briefly, biotin-labeled miR-552-3p WT/MUT plasmids (50 nM each) were transfected into INS-1E cells for 24 h. Afterword, the cells were harvested rinsed off and incubated with M-280 streptavidin magnetic beads (Sigma). Then, biotin-bound proteins were pull down, extracted, and enrichment of circPIP5K1A was detected by RT-qPCR analysis while an antagonistic miR-552-3p probe was set up as an NC [14].

### 2.13 RNA binding protein immunoprecipitation (RIP) assay

RNA binding protein immunoprecipitation (RIP) assay was executed by using Magna RNA RIP Kit (Merck KGaA, Darmstadt, Germany) by following the manufacturer’s protocol. INS-1E cells were lysed by using RIP lysis buffer (Beyotime) and incubated with magnetic beads conjugated with Argonaute-2 (Ago2, Millipore, MA, USA) or control immunoglobulin G (IgG, Millipore) at 4ʹC overnight. Detachment of the complex was with proteinase K (Sigma-Aldrich), and the bound RNA was identified by RT-qPCR [14].

### 2.14 Statistical analysis

The data were statistically analyzed via SPSS 22.0 and presented as the mean ± standard deviation (SD) of three independent experiments. One-way analysis of variance (ANOVA) and Student’s t-test were applied to compare the differences the among groups. Pearson’s correlation was conducted to analyze the link among circPIP5K1A, miR-552-3p, and JAK1, and the value *P* < 0.05 was considered as statistically significant.

## 3 Results

Studies have suggested that miR-552-3p is a novel inhibitor [18] and is involved in numerous momentous biological processes such as immune modulation, differentiation, and apoptosis [20]. However, Arianna Mazzoli *et al*. [21] found that decreased expression of JNK1 resulted in improved glucose tolerance in db/db mice by downregulating the dysfunctional β cells in diabetes. However, whether miR-552-3p controls the β cells of T2DM through the JAK1-STAT3 pathway still needs to be addressed. The aim to conduct this study was to evaluate the molecular mechanism of circPIP5K1A in β cells of T2DM, and how it regulates the miR-552-3p with JAK1-STAT3 pathway.

### 3.1 Elevated circPIP5K1A and JAK1, but reduced expression of miR-552- in the serum sample of T2DM patients

**Figure 1.**
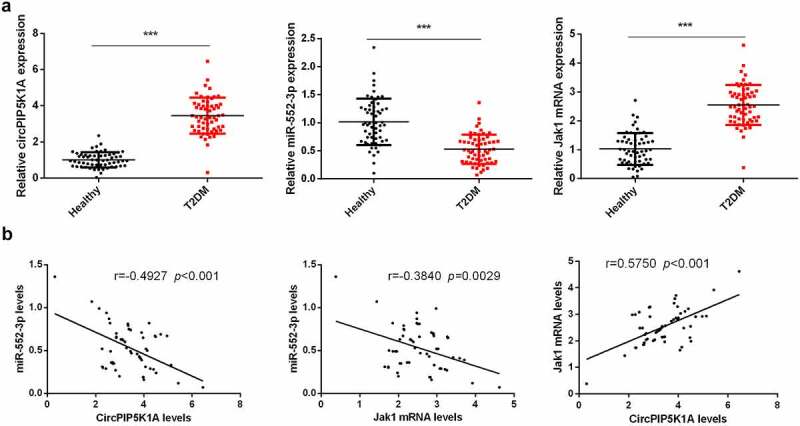
Elevated circPIP5K1A and JAK1 but reduced miR-552-3p are presented in the serum sample of T2DM patients.

Relative expression of circPIP5K1A, JAK1, and miR-552-3p in blood samples of T2DM patients was measured, while healthy individual’s blood samples were used as control. Our findings manifested that level of circPIP5K1A and JAK1 in the serum of T2DM patients was significantly upregulated and miR-552-3p level was apparently reduced as compared to the control (Figure 1a). We further suggested that serum circPIP5K1A with miR-552-3p and miR-552-3p with JAK1 were negatively linked, while circPIP5K1A was positively correlated with JAK1 (Figure 1b). These results provided the first evidence that elevated circPIP5K1A was vital in the development of T2DM.

A: RT-qPCR to detect circPIP5K1A, miR-552-3p, and JAK1 in the serum of T2DM patients, n = 58; ***P < 0.001; B: The analysis of liner link between circPIP5K1A and miR-552-3p, miR-552-3p and JAK1, circPIP5K1A and JAK1 in T2DM serum was carried out by Spearman’s correlation coefficient.

### 3.2 Downregulated circPIP5K1A represses the inflammation, oxidative damage, and apoptosis of INS-1 β cell induced via glucolipotoxicity

**Figure 2.**
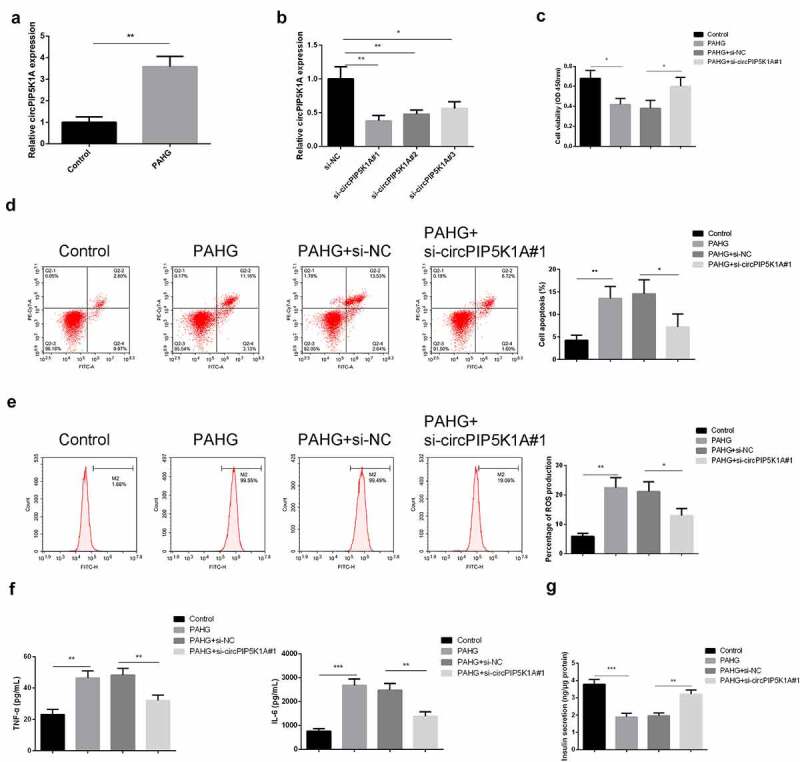
Downregulated circPIP5K1A restrains the inflammation, oxidative damage, and apoptosis of INS-1 β cell induced via glycolipid toxicity.

Glucolipotoxicity injury of pancreatic β cells is a key pathological feature of T2DM. In order to investigate the glucolipotoxicity-induced circPIP5K1A expression in INS-E1 β cell, INS-1E cells were exposed to PAHG conditions, and detection of circPIP5K1A was conducted. The results manifested that circPIP5K1A was enhancive under PAHG conditions (Figure 2a). To confirm our findings that PAHG was directly associated with increased expression of circPIP5K1A, transfection of si-circPIP5K1A inINS-1E cells has been performed. Outcomes suggested that si-circPIP5K1A#1 manifested higher silencing efficiency (Figure 2b), so si-circPIP5K1A#1 was selected for subsequent experiments. Our findings suggested that exposure of INS-1E cells to PAHG leads to increased rate of the cell proliferation, and silencing of circPIP5K1A can reverse the damaging effects of PAHG in INS-1E cells (Figure 2c-D). The results of FACS analysis of ROS identified that silencing of circPIP5K1A have the potential to reverse the effects of PAHG-induced oxidative damage in INS-1E cells (Figure 2e). Detection of the inflammatory factors clarified that after INS-1E cells’ exposure to PAHG, TNF-α and IL-6 was elevated, while silencing circPIP5K1A could reduce inflammation reaction (figure 2f). After measuring insulin secretion, it came out that PAHG introduction lessened the insulin secretion response of INS-1E cells to 16.7 mM glucose by about 50%, while depressive circPIP5K1A could partially turn around this response (Figure 2g). The results clarified that silencing of circPIP5K1A could reduce the toxic effect of glycolipid toxicity on INS-1 β cell.

A/B: RT-qPCR to detect circPIP5K1A; C: CCK-8 to detect INS-1E cell viability and each bar represent the mean ± standard deviation of three independent experiments; D, E: Flow cytometry to detect INS-1E cell apoptosis and measure cellular ROS. Each bar represented the mean ± standard deviation of three independent experiments in which *value show significant difference as compared to the control (*P < 0.05). F, G: ELISA to detect inflammatory factors TNF-α and IL-6 in INS-1E cells and measure secreted insulin. Cells were transfected with 50 nmol of oligonucleotides and incubated for 48 hours before subsequent assays. *P < 0.05, **P < 0.01, ***P < 0.001.

### 3.3 CircPIP5K1A adsorbs miR-552-3p in INS-1E cells

**Figure 3.**
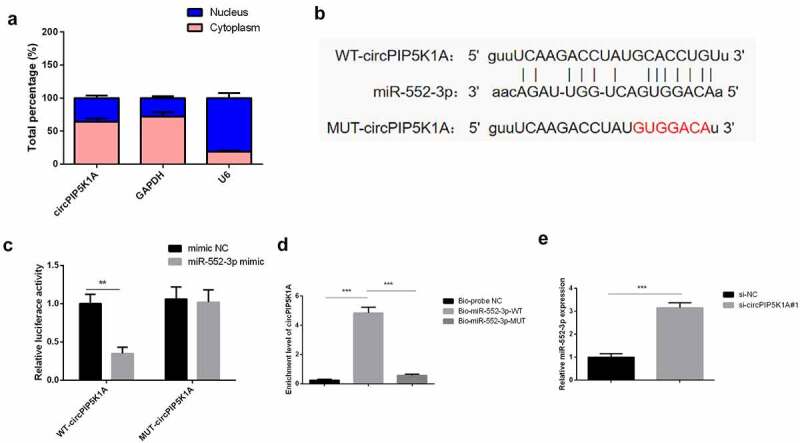
CircPIP5K1A adsorbs miR-552-3p in INS-1E cells.

In order to further understand how circPIP5K1A plays a key role in INS-1E cells, a sub-cellular hierarchical analysis was first performed to detect its cellular location in INS-1E cells. In line with the results, circPIP5K1A seemed to be majorly situated in INS-1E cells’ cytoplasm (Figure 3a). Therefore, it was speculated that circPIP5K1A might post-transcriptionally control the gene via acting as a competitive endogenous RNA (ceRNA) in INS-1E. Through starbase (http://starbase.sysu.edu.cn/) was found a binding site of circPIP5K1A with miR-552-3p (Figure 3b). Moreover, upregulated miR-552-3p apparently reduced the luciferase activity of pmirGLO-circPIP5K1A-WT, while no apparent transformations in the luciferase activity of MUT existed in disparate groups (Figure 3c). An experiment was conducted to verify that circPIP5K1A could be applied as a ceRNA to adsorb miR-552-3p. It was emphasized that the enrichment of circPIP5K1A in the Bio-miR-552-3p-WT was clearly elevated vs. that of Bio-miR-552-3p-MUT and Bio-probe NC groups (Figure 3d). And depression of circPIP5K1A was available to elevate miR-552-3p (Figure 3e). The above results emphasized that circPIP5K1A modulated miR-552-3p via competitively combining with miR-552-3p.

A: Nuclear and cytoplasmic separation experiments to testify circPIP5K1A was mainly situated in the cytoplasm; B: Bioinformatics website starbase to forecast the binding site of circPIP5K1A and miR-552-3p; C: Dual luciferase verification of circPIP5K1A and miR-552-3p’s binding; D, E: RNA pull-down to examine the enrichment of miR-552-3p on circPIP5K1A and miR-552-3p after depressive circPIP5K1A. Cells were transfected with 50 nmol of oligonucleotides and incubated for 48 hours before subsequent assays. Graph presented mean ± standard deviation results of three independent experiment *P < 0.05, **P < 0.01, ***P < 0.001.

### 3.4 Silencing of miR-552-3p reverse the depletive effects of circPIP5K1A by reducing glucolipotoxicity on INS-1 β cell

**Figure 4.**
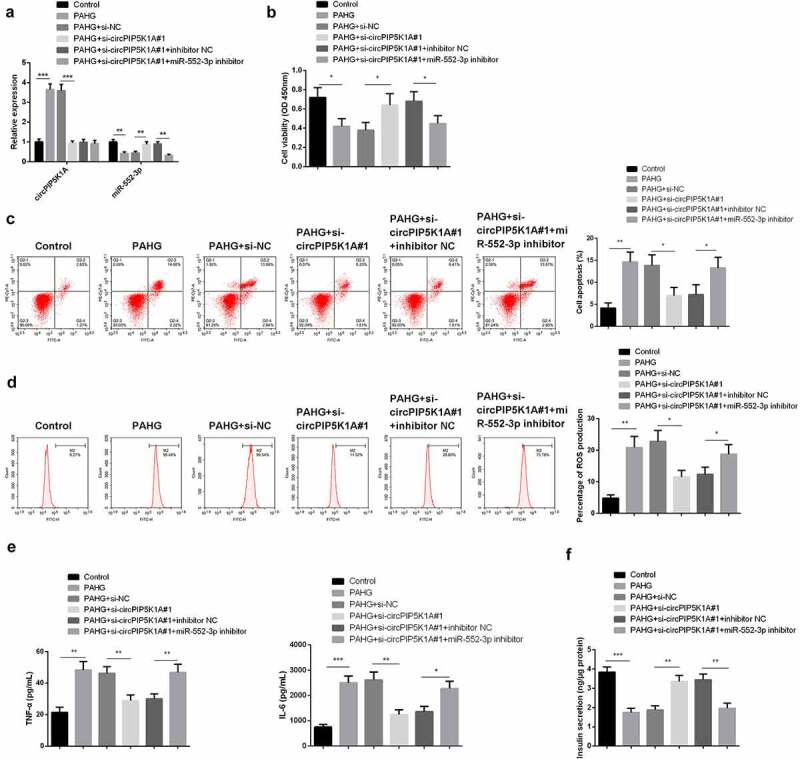
Downregulating miR-552-3p turns around that dissipative circPIP5K1A reduces the toxic effect of glycolipid toxicity on INS-1 β cell.

For clarifying whether circPIP5K1A’s reduction of glucolipotoxicity in INS-1E cell is depended on the modulation of miR-552-3p, application of si-circPIP5K1A#1 + miR-552-3p inhibitor was to transfect INS-1E cells. The analysis manifested that miR-552-3p expression was reduced under PAHG conditions, while silencing of circPIP5K1A restored miR-552-3p (Figure 4a). Additionally, in PAHG-induced INS-1E cells, restrained miR-552-3p could reverse the motivation in cell proliferation via repressive circPIP5K1A (Figure 4b-C). Detecting cellular inflammatory factors manifested in PAHG-induced INS-1E cells, downregulation of circPIP5K1A could restrain ROS, TNF-α, and IL-6, while simultaneously reducing circPIP5K1A and miR-552-3p could reverse this effect (Figure 4d-E). Insulin secretion measurement revealed that depressive circPIP5K1A and miR-552-3p clearly restored the insulin secretion affected via si-circPIP5K1A#1 transfection (figure 4f). The above results clarified in PAHG-induced INS-1E cells, restrained miR-552-3p could reverse the mitigation of declined circPIP5K1A on cell inflammation, oxidative damage, and apoptosis induced by glucolipotoxicity.

A: RT-qPCR to test circPIP5K1A and miR-552-3p; B: CCK-8 to examine INS-1E cell viability; C, D: Flow cytometry to detect INS-1E cell apoptosis and measure cellular ROS. Each bar represented the mean ± standard deviation of three independent experiments in which *value show significant difference as compared to the control (*P < 0.05). E, F: ELISA to detect inflammatory factors of TNF-α and IL-6 in INS-1E cells and measure secreted insulin. Cells were transfected with 50 nmol of oligonucleotides and incubated for 48 hours before subsequent assays. Graph presented the mean ± standard deviation of three independent experiments in which *value show significant difference as compared to the control (*P < 0.05) *P < 0.05, **P < 0.01, ***P < 0.001.

### 3.5 JAK1 performs as a targeted mRNA downstream of miR-552-3p

**Figure 5.**
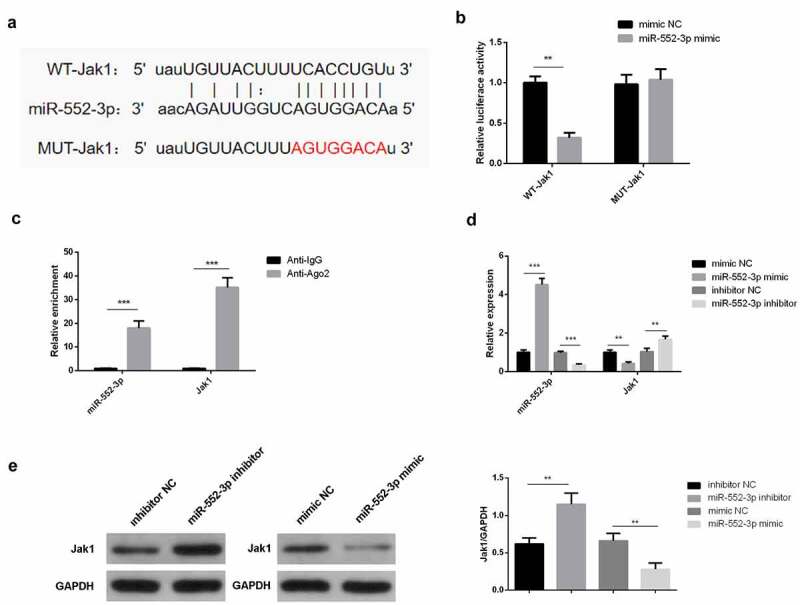
JAK1 acts as a targeted mRNA of miR-552-3p.

Through the starbase website was discovered a binding site of miR-552-3p with JAK1 (Figure 5a). It has been found that the combination of JAK1 with miR-552-3p did exist (Figure 5b), which was consistent with the results of the RIP assay (Figure 5c). Meanwhile, miR-552-3p promotes the expression of JAK1 while miR-552-3P inhibitor downregulated the JAK1 (Figure 5d-E). In short, miR-552-3p directly targeted JAK1 and negatively modulated its expression.

A: Bioinformatics website starbase to forecast the binding site of miR-552-3p and JAK1; B, C: Dual luciferase verification and RIP detection of miR-552-3p and JAK1’s binding; D, E: RT-qPCR and WB to detect JAK1 after elevated or depressive miR-552-3p. Cells were transfected with 50 nmol of oligonucleotides and incubated for 48 hours before subsequent assays. Graph presented the mean ± standard deviation of three independent experiments in which *value show significant difference as compared to the control (*P < 0.05) *P < 0.05, **P < 0.01, ***P < 0.001.

### 3.6 MiR-552-3p restrains the inflammation, oxidative damage, and apoptosis of INS-1 β cell induced via glucolipotoxicity by targeting the JAK1-STAT3 pathway

**Figure 6.**
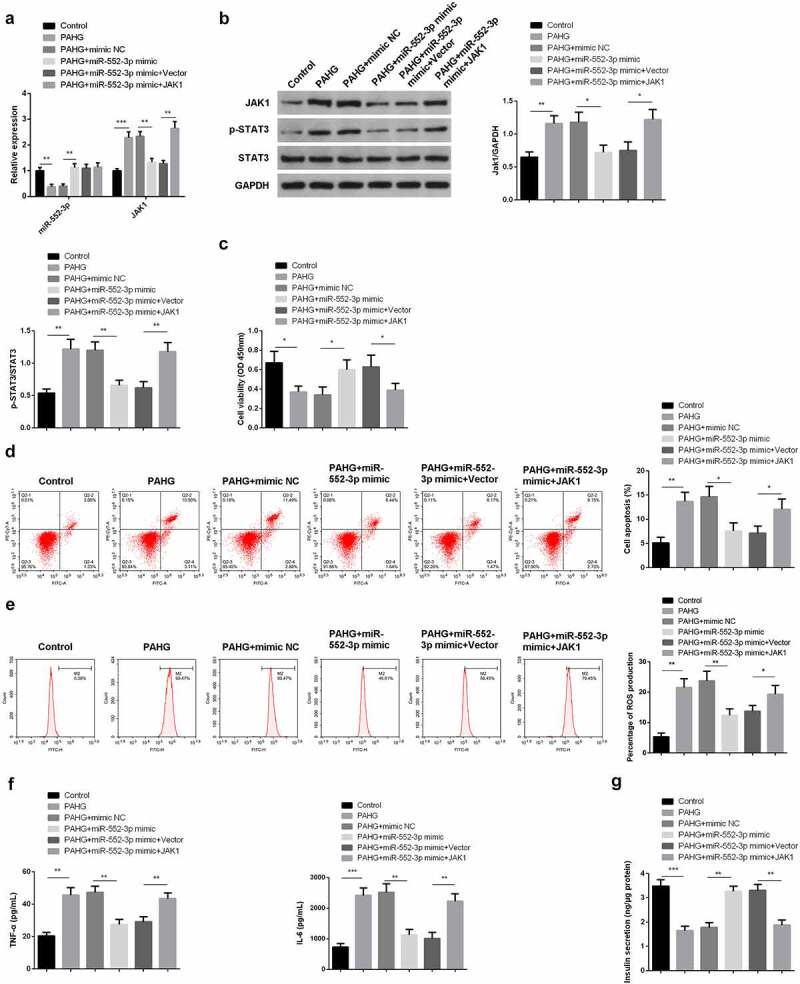
MiR-552-3p refrains the inflammation, oxidative damage, and apoptosis of glycolipid toxicity-stimulated INS-1 β cells via targeting the JAK1-STAT3 pathway.

For further clarifying the function and molecular mechanism of miR-552-3p in glucolipotoxicity-induced INS-1 β cell, transfection of miR-552-3p mimic with JAK1 elevation vectors was into PAHG-treated INS-1E cells. Meanwhile, detecting miR-552-3p and JAK1-STAT3 pathway was conducted. The results affirmed that co-transfection of miR-552-3p mimics and JAK1 overexpression plasmid significantly reverse the effects of miR-552-3p mimics by upregulating the expression of JAK1 and leads to the phosphorylation of STAT3 (Figure 6a-B). Additionally, in PAHG-induced INS-1E cells, miR-552-3p could induce the motivation in cell advancement, which was turned around via JAK1 (Figure 6c-D). Detecting cellular inflammatory factors manifested in PAHG-induced INS-1E cells, miR-552-3p repressed ROS, TNF-α, and IL-6, while simultaneous upregulation of miR-552-3p and JAK1 reversed this effect (Figure 6e-F). Measuring insulin secretion revealed that miR-552-3p facilitated insulin secretion, while JAK1 was opposite (Figure 6g). The above results clarified that in PAHG-induced INS-1E cells, miR-552-3p reduced the toxic effect of glucolipotoxicity on INS-1E cells via targeting the JAK1-STAT3 pathway.

A, B: RT-qPCR and WB to detect miR-552-3p, JAK1, p-STAT3, STAT3; C: CCK-8 to detect INS-1E cell viability; D, E: Flow cytometry to detect INS-1E cell apoptosis and measure cellular ROS; F, G: ELISA to detect inflammatory factors TNF-α and IL-6 in INS-1E cells and measure secreted insulin. Cells were transfected with 50 nmol of oligonucleotides and incubated for 48 hours before subsequent assays. Graph presented the mean ± standard deviation of three independent experiments in which *value show significant difference as compared to the control (*P < 0.05) *P < 0.05, **P < 0.01, ***P < 0.001.

## 4 Discussion

Progressive decrease in pancreatic β cells function may lead toward chronic diseases such as T2DM [5]. A study has also manifested that elevated level of glucose and fatty acid are the point of concern for increasing ratio of T2DM-related deaths [25]. Owing to numerous inconsistent reports the exact mechanism of glucolipotoxicity in pancreatic β cells is still remaining unclear. Hence, the aim of this study was to clarify the mechanism in which circPIP5K1A controlled glucolipotoxicity in INS-1E cells through the miR-552-3p/JAK1-STAT3 axis.

Although its reported that circRNAs has played a significant role in the development of certain life-threatening diseases such as cancer and diabetes [26,27]. Recently, Wang *et al*. reported that 1,020 elevated and 902 reduced circRNAs are identified in β cells introduced with cytokines, and repressive circRNA 000286 and 01727 in β cells can stimulate apoptosis by affecting insulin biosynthesis and secretion [28]. Refrained hsa_circ_0054633 prevents HG-induced human pancreatic β-cell apoptosis and impaired insulin secretion via controlling the miR-409-3p/caspase-8 axis [29]. The circLRP6/miR-9-5p/PRMT axis impacts the cell proliferation, insulin stimulation, and oxidative stress of INS-1E cells attacked by streptozotocin (STZ) [30].

Till date, CDR1, cerebellum degeneration-related antigen 1, also termed as ciRS-7, is most known diabetes-related circRNA, and it is produced from a natural antisense strand of CDR1. Studies have shown that increased expression of CDRI may lead to the elevated secretion level of insulin, and this increased insulin secretion subsequently repaired the dysfunctional β cells [27]. miR-7 is a powerful inhibitor [27] of CDR1and stimulate the proliferation of β cells and increased the secretion of insulin via encouraging the expression of Pax6 and Myrip genes [27]. On the other hand, in vivo studies in transgenic mice have shown that increased expression of miR-7a resulted in decreased level of insulin secretion in β cell followed by dedifferentiation of β cells [31]. Stoll et al. investigated the expression of circHIPK3 and ciRS-7/CDR1 and found that decreased expression of circHIPK3 and ciRS-7/CDR1 was reduced in the islets of diabetic transgenic mice [32]. While, silencing of circHIPK3 and ciRS-7/CDR1 in the small patches of normal mice caused impaired insulin secretion, poor rate of β-cell proliferation, and lower survival rate, indicating a possible role of transformed expression of circHIPK3 and ciRS-7 in the development of diabetes mellitus [32]. These findings goes parallel with those of Cao et al., and it stated that the decreased expression of circHIPK3 in diabetic patients was significantly associated with high glucose-treated HUVECs and primary aortic endothelial cells (HAECs) [33]. These findings further suggested that circular RNAs might be possible remedial targets in diabetes.

In this study, increased expression of circPIP5K1A has been found in serum samples of T2DM patients as compared to controls. However, studies have shown that increased expression of circPIP5K1A is involved in upregulation of cell proliferation by modulating the cell cycle and apoptosis [22]. On the one hand, in our study, we have found that glucolipotoxicity-induced downregulation of circPIP5K1A repressed the inflammation, oxidative damage, and apoptosis of INS-1E cells. These findings assured that circPIP5K1A might be crucial in the pathogenesis of T2DM.

MiRNAs always interact with circRNAs and play a bridge role in perfecting disease signaling pathways [34]. Circ_001209 worsens diabetic retinal vascular dysfunction via mediating miR-15b-5p/COL12A1 [35]. Silencing of circANKRD36 targets miR-145 via XBP1 to restrain STZ-caused insulin resistance and inflammation in diabetic rats [23]. CircEIF4G2 aggravates the renal fibrosis of diabetic nephropathy through sponging miR-218 [36]. Meanwhile, the study clarified that circPIP5K1A motivated the cell inflammation, oxidative damage, and apoptosis induced by glucolipotoxicity via absorbing miR-552-3p. Moreover, miR-552-3p targeted JAK1 3ʹ UTR and negatively controlled its expression. A former study emphasizes that JNK1 ablation mitigates glucose tolerance in db/db mice via lessening the loss of functional β cells in db/db obesity-driven diabetes [37]. Additionally, T cell protein tyrosine phosphatase non-receptor type 2 can help decline periodontal inflammation in T2DM via refraining the dephospho-protein substrate in the JAK1/STAT3 pathway [38]. Similarly, our results also demonstrated that miR-552-3p restrained the inflammation, oxidative damage, and apoptosis of glucolipotoxicity-stimulated INS-1 β cell via targeting the JAK1-STAT3 pathway. Hence, it can be concluded that depressive circPIP5K1A via miR-552-3p/JAK1-STAT3 axis reduces the mechanism of INS-1E cytotoxicity induced by glucolipotoxicity.

## 5 Conclusions

In general, in T2DM tissues and cells, circPIP5K1A and JAK1 were upregulated, but miR-552-3p was downregulated. Repressing circPIP5K1A via miR-552-3p/JAK1-STAT3 axis restrains the inflammation, oxidative damage, and apoptosis of glycolipid toxicity-stimulated INS-1E cells. CircPIP5K1A is expected to become a latent clinical biomarker of T2DM, offering new ideas for further understanding of the molecular pathology of T2DM. It is of great significance for formulating specific targeted therapy strategies for T2DM.

## Highlights

1. Elevated circPIP5K1A and JAK1 but reduced miR-552-3p were presented in the serum of T2DM patients.

2. Downregulating circPIP5K1A reduced the toxic effect of glucolipotoxicity on INS-1 β cells.

3. CircPIP5K1A adsorbed miR-552-3p, which targeted JAK1.

4. CircPIP5K1A modulated INS-1E cell dysfunction induced via glucolipotoxicity through miR-552-3p/JAK1-STAT3 axis.
